# Investigation on Pathological Aspects, Mode of Transmission, and Tissue Tropism of *Antheraea proylei* Nucleopolyhedrovirus Infecting Oak Tasar Silkworm

**DOI:** 10.1093/jisesa/ieac057

**Published:** 2022-10-07

**Authors:** Diksha Khajje, Sinam Subharani Devi, Gangavarapu Subrahmanyam, Jun Kobayashi, Vankadara Sivaprasad, Olle Terenius, Kangayam M Ponnuvel

**Affiliations:** Genomic Division, Seri biotech Research Laboratory, Carmelaram Post, Kodathi, Bangalore 560035, India; Department of Biotechnology, School of Sciences, Jain University, Bangalore 560027, India; Regional Sericultural Research Station, Imphal, Manipur 795002, India; Genomic Division, Seri biotech Research Laboratory, Carmelaram Post, Kodathi, Bangalore 560035, India; Graduate School of Sciences and Technology for Innovation, Yamaguchi University, 1677-1, Yoshida, Yamaguchi 753-8515, Japan; Genomic Division, Seri biotech Research Laboratory, Carmelaram Post, Kodathi, Bangalore 560035, India; Department of Cell and Molecular Biology, Uppsala University, Box 536, SE-751 23 Uppsala, Sweden; Genomic Division, Seri biotech Research Laboratory, Carmelaram Post, Kodathi, Bangalore 560035, India

**Keywords:** Tiger band disease, oak tasar silkworm, vertical transmission, tissue tropism, sericulture

## Abstract

The temperate oak tasar silkworm, *Antheraea proylei,* is frequently infested with *Antheraea proylei* nucleopolyhedrovirus (AnprNPV) causing tiger band disease. This disease is one of the key factors that obstructs production and productivity of oak tasar sericulture. The current study aimed to investigate the pathogenicity of AnprNPV, its mode of transmission, and detection of AnprNPV in different tissues. Transmission electron micrographs of AnprNPV showed single rod-shaped bodies and occlusion derived virus (ODV) enclosed within multiple envelopes. The infecting AnprNPV displayed tissue tropism with higher copy numbers detected in the insect fat body and ovary. The virus was observed to multiply in all developmental stages of the silkworm such as egg, larva, pupa, and moth, confirming its ability to spread throughout the silkworm lifecycle. Baculovirus isolated from infected *A. proylei* showed cross-infectivity in other Saturniidae wild silkworm species such as *Antheraea pernyi*, *A. frithi*, and *Samia ricini*, widening their probable host range for infection. Baculoviruses generally display a horizontal mode of transmission, mainly through ingestion of occlusion bodies (OBs); however, the present study revealed a trans-ovum vertical mode of transmission in addition to a horizontal mode. The observations made in this study aid a detailed understanding of the tiger band disease and its causative pathogen AnprNPV, which will support future studies and disease management in oak tasar sericulture.

The oak tasar silkworm, *Antheraea proylei* Jolly (Lepidoptera: Saturniidae), is one of the most economically important and commercially exploited wild silkworms reared for oak tasar silk production (Nath et al. 2021). The members found under the genus *Antheraea* occupy different ecological niches, ranging from tropical to temperate along with transitional zones. Among the several species of oak tasar silkworms, *A. proylei* is the most prominent, viable, and commercially exploited species in India. *A. proylei* is an inter-specific hybrid of *A. roylei* Moore (Lepidoptera: Saturniidae) of India and *A. pernyi* Guérin-Méneville (Lepidoptera: Saturniidae) of China. Oak tasar cultivation is followed throughout the sub-Himalayan belt extending from Jammu and Kashmir in the north to Manipur in the Far East (Shantibala et al. 2018). Oak tasar silkworms have been reared on a wide variety of host plants such as *Quercus floribunda*, *Q. himalayana*, *Q. semecarpifolia*, *Q. serrata*, and *Q. leucotrichophora*. The oak flora plays a significant role in enhancing the economy of the hill tribes as well as the economically challenged communities residing in the foothills of Himalayas. Cultivation of oak and *A. proylei* thus provide an alternate source of income to enhance the livelihood of small-scale farmers and several tribal communities. Like other lepidopterans, Oak tasar silkworm has four developmental stages such as egg, larva, pupa, and adult moth. The female moth emerges in the early spring (February) and immediately mates with a male. After egg laying, it takes approximately 10 d until eclosion. The larval stages are the only feeding stage of the silkworm. During late spring (March–April), which is the favorable commercial rearing season, it completes 4 molts and enters pupal diapause for the next 9 mo.

Since oak tasar silkworms are non-domesticated and are reared outdoors in the wild, they are susceptible to various microbial diseases including microsporidiosis, virosis, bacteriosis, and mycosis (Shantibala et al. 2018). Tiger band disease is one of the most common and dreadful diseases of oak tasar silkworms and causes severe losses to the sericulture industry. Variations in temperature and high humidity are the main predisposing factors for the disease in *A. proylei* larvae (Bergren and Kading 2018). The extent of damage due to tiger band disease was found to be approximately 70–80% of *A. proylei* larval and pupal mortality (Shantibala et al. 2018). Tiger band diseased larvae show poor growth and development along with loss of appetite. The disease is characterized by the appearance of black bands/stripes across the body of the silkworm (hence, the name tiger band disease, Supp Fig. 1 [online only]) (Aragão-Silva et al. 2016). Other symptoms include shrinkage and softening of the body eventually leading to death (Shantibala et al. 2018). Field observations indicate that the disease is primarily transmitted horizontally through contaminated leaves and rearing appliances.

The causal organism of tiger band disease has been identified as *A. proylei* nucleopolyhedrovirus (AnprNPV), which belongs to Alphabaculovirus in the Baculoviridae family (Shantibala et al. 2018). Baculoviruses are insect specific occluded viruses with rod shaped nucleocapsids. Transmission of baculoviruses occurs through the oral or ‘per os’ route when insects consume occlusion bodies (OBs) through food. Food particles contaminated with OBs enter the insect midgut where they are exposed to higher alkaline pH (pH 10–11). The alkalinity of the insect midgut triggers the dissolution of OBs and the release of occlusion derived virions (ODVs) into the midgut lumen. These released virus particles are defined as ODVs (Slack and Arif 2006). It was reported that ODVs are released from OBs within 12 min post entry into the insect midgut (Adams and McClintock 1991). Genetically, these baculoviruses have a double stranded super coiled circular DNA genome with a molecular size ranging from 80 to 180 kb (Shantibala et al. 2018). Based on the phylogenetic information of 29 core genes, biological and morphological characteristics, the family Baculoviridae was subdivided into four genera: Alphabaculovirus (lepidoperan-specific nucleopolyhedroviruses), Betabaculovirus (lepidopteran specific granuloviruses), Gammabaculovirus (hymenopteran-specific nucleopolyhedroviruses) and Deltabaculovirus (Diptera-specific baculoviruses) (Jehle et al. 2006). Over 600 species of insects have been reported to be infected with baculoviruses, including those belonging to the insect orders Lepidoptera, Hymenoptera, and Diptera (Bergren and Kading 2018). The genomes of AnprNPV (Accession no LC375539) and other baculoviruses (Accession no LC375537, LC375538, and DQ486030) infecting Saturniidae silk moths share a high degree of similarity, implying they have evolved from a common ancestry origin. However, a detailed comparative genomics of baculoviruses is necessary to understand the evolution of genes involved primarily in replication, transcription, and other structural protein genes (Hill and Unckless 2017).

With this background, the current study was designed to investigate AnprNPV pathogenicity, mode of transmission, and tissue tropism during infection of *A. proylei*. Enhanced knowledge on the disease would help us to design appropriate disease control strategies for improving silk production which will lead to increased remuneration of the tribal community associated with the oak tasar silk industry.

## Materials and Methods

### Virus Studies

#### Collection of Oak Tasar Silkworms

Fifth instar *A. proylei* larvae infected with AnprNPV were collected from the sericulture fields of Regional Sericulture Research Station (RSRS), Imphal, Manipur, India, during one autumn season (October, 2018). A total of 500 infected larvae and 500 healthy larvae were collected for the study. Infected larvae were characterized by dark tiger-like stripes across the body of the silkworm, while healthy silkworms did not show any disease symptoms. The sampling site is located in extreme eastern India and has a distinct subtropical climate characterized by hot, humid summers and dry winters. During the autumn season, the minimum temperature was 20.4°C and the maximum temperature was 34.9°C, while the humidity range was 55–85%.

#### Isolation of Viral Polyhedra

Viral polyhedral bodies were isolated from the infected oak tasar silkworms using a method previously described (Shantibala et al. 2018). In brief, the infected silkworm samples were placed in dark amber colored bottles with distilled water and allowed to putrefy for 15 d. The putrefied samples were treated with ampicillin and chloramphenicol at a conc. of 50 µg/ml before further processing. The treated cadavers were filtered through cheese cloth and subjected to centrifugation at 100 g for 2 min to remove floating debris. The supernatant was collected and further centrifuged at 4,000 g for 15 min to pellet polyhedra. The pellet was washed thrice at room temperature with 0.1% SDS to remove any floating debris. Subsequent centrifugations were performed at 4,000 g for 10 min. The concentration of the purified viral polyhedra was determined using a hemocytometer.

#### Transmission Electron Microscopy

Transmission electron microscopy (TEM) was used to examine the structure and morphology of virions as per the protocol of Kumar et al. 2011. The purified viral polyhedra/OB sample was pelleted by centrifugation at 5,000 g for 5 min, resuspended with 3% glutaraldehyde, and fixed overnight at room temperature. The samples were concentrated in 1 ml of 0.1M cacodylate buffer and post-fixed with 2% osmium tetroxide (OsO_4_) for 2 hr at 4°C. Samples were dehydrated using a series of graded ethanol from 70% to 100% and stained for 1 hr with 2% uranyl acetate. The samples were washed with propylene oxide to remove residual ethanol from dehydration. The samples were further embedded in araldite resin and polymerized for 48 hr at 60°C. After polymerization, samples were sectioned, transferred to TEM grids, and stained with lead acetate. The resulting grids were observed under a transmission electron microscope at 80 kV (JEOL JEM 1400 plus, JEOL Ltd., Tokyo, Japan).

### Pathogenicity and Mode of Transmission of AnprNPV

#### Infection Studies with AnprNPV

Lethal Concentration 50 (LC50) of AnprNPV in *A. proylei* was determined. In brief, 100 µl of concentrations of AnprNPV polyhedral suspensions ranging from 2.2 × 10^8^ OB/ml to 2.2 × 10^2^ OB/ml were smeared on a disc of 4 cm diameter of *Q. serrata* leaves (the primary food plant of *A. proylei*) and air dried (Reeta et al. 2008). Before conducting the experiment, *Q. serrata* leaves were surface sterilized with 70% alcohol to remove any microbial contamination. Three replicates of 30 third instar *A. proylei* larvae (collected from RSRS, Imphal) were provided food on the air-dried virus smeared leaves. As controls, *A. proylei* larvae were fed with *Q. serrata* leaves with the same volume of sterile water. On complete feeding of the leaf disc, the larvae were transferred to fresh *Q. serrata* twigs inserted in bottles and reared under laboratory conditions. The larvae were reared at 24°C and 70–75% relative humidity under a 16:8 light: dark regime. Larval mortality was recorded daily and the LC50 value of AnprNPV in *A. proylei* was determined by Probit analysis with Finney’s Probit analysis spreadsheet calculator (https://probitanalysis.wordpress.com/).

#### Source of Infection and Surface Disinfection

To determine the source of infection of AnprNPV, female *A. proylei* moths collected from RSRS, Imphal sericulture fields were allowed to mate. After which the female moths were placed in separate nylon bags for egg laying for a period of 4–5 d. After oviposition, the female moths were tested for the presence of the virus with PCR using primers targeting the *Anpr*53 gene associated with late infection and capsid proteins (Supp Table 1 [online only]).

Upon confirmation of an infection, a total of 100 eggs were collected from a single AnprNPV infected female moth for further processing. 50 eggs were treated for surface disinfection by immersing the eggs in 0.2% sodium hypochlorite for 20 min followed by subsequent wash under running tap water to remove the residual disinfectant solution. Another 50 eggs from the same infected mother moth were used directly as a control without any surface disinfection for further analysis. All the egg samples (both treated and untreated) were washed with lysis buffer (200 mM Tris, 300 mM NaCl, 25 mM EDTA with 20% SDS, pH 7.4). The wash buffer was used in subsequent steps of DNA isolation. These treated and untreated eggs were further allowed to hatch and hatched larvae were checked for the presence of virus through PCR analysis.

#### DNA Isolation From Eggs and Hatched Larvae

The isolation of DNA from eggs and hatched larvae was performed by crushing the samples using mortar and pestle in 200 µl of lysis buffer (Gupta et al. 2022). The crude material was mixed thoroughly and centrifuged at 3,000 × *g* for 5 min at 15°C. The pellet and supernatant were separated and 200 µl of lysis buffer was added along with 2 µl of Proteinase-K (200 μg/ml), followed by the incubation at 65°C for 1 hr. An equal volume of PCI (phenol chloroform Isoamyl alcohol, 25:24:1) was added to the supernatant followed by centrifugation at 11,500 × *g* for 20 min at 4°C. The upper aqueous layer was transferred to another tube and an equal volume of chloroform was added, centrifuged at 11,500 × *g* for 20 min. To the supernatant, 50 µl 3M sodium acetate and 3 volumes of chilled 100% absolute alcohol was added and mixed by inversion. After centrifugation at 11,500 × *g* for 20 min at 4°C, the pellet was dried before dissolving in 50 µl of sterile nuclease free water.

#### DNA Isolation From Viral Particles

To isolate viral DNA from the purified polyhedra, 1ml of purified polyhedra suspension was adjusted to a concentration of 5 × 10^9^/ml to 1 × 10^10^/ml. To this 500 µl of 0.3M sodium carbonate was added (Shantibala et al. 2018). This was incubated for 2 hr on a shaker incubator (Innova 3230, New Brunswick Scientific, Enfield, CT) at 150 × *g* at 37°C followed by centrifugation at 12,500 × *g* for 10 min at 4°C. To the supernatant 2 µl of proteinase K was added along with 500 µl of lysis buffer. The mixture was incubated at 56°C in a water bath (GeNei, Bangalore, India) for 2 hr. An equal volume of PCI was added followed by centrifugation at 10,000 × *g* for 20 min at 4°C. The upper aqueous layer was transferred to another tube and an equal volume of chloroform was added, followed by centrifugation at 10,000 × *g* for 20 min at 4°C. The resulting upper layer was taken into a fresh tube and 50 µl of 3M sodium acetate along with 3 volumes of chilled 100% alcohol were added. DNA was pelleted by centrifugation at 10,000 × *g* for 20 min. The pellet was dried and dissolved in 100 µl of sterile water, and the DNA obtained was stored at 4°C for future use. The DNA concentration was determined using a NanoDrop 2000C (Thermo Fisher Scientific, Waltham, MA).

### Tissue Tropism and Cross Infection of AnprNPV

#### DNA Isolation From Tissues

AnprNPV infected fifth instar A*. proylei* larvae showing symptoms of restlessness, sluggishness, starvation, and soft loose cuticle with the appearance of black bands on the larval body segments were collected from RSRS, Manipur, India. About 100 mg of each tissue such as midgut, fat body, trachea, Malpighian tubules, ovary, testis, pupa, egg inner content, moth, and silk gland was dissected for genomic DNA isolation as described in our previous study (Gupta et al. 2022).

Silkworm samples from different developmental stages such as egg, larva, pupa, and moth, were also collected from the sericulture fields and the DNA was isolated from ~100 mg per tissue to check for the presence of the virus using PCR. The DNA was isolated as per the procedure described in Esvaran et al. 2020.

#### Cross-Infection of AnprNPV

The cross-infectivity of AnprNPV in other wild silkworms was studied by inoculating 3rd instar silkworm (3 replicates; 30 larvae/replication of each spp.) of *S. ricini*, *A. pernyi* and *A. frithi* with AnprNPV. Various concentrations of 100 µl of AnprNPV polyhedral suspensions (2.2 × 10^8^ OB/ml–2.2 × 10^2^ OB/ml) were prepared and applied on leaves which were air dried before being fed to the silkworms. For a control setup, virus un-inoculated 3rd instar silkworm of each spp. (*S. ricini* [Lepidoptera: Saturniidae], *A. pernyi*, and *A. frithi* [Lepidoptera: Saturniidae]) were reared on *Q. serrata* leaves. The silkworms were transferred to twigs inserted in bottles and reared indoors. The mortality of the silkworms was observed and recorded on different days post-infection. The cultures were maintained appropriately by routine feeding and cleaning on a daily basis to remove the excreta. The larvae were reared at 24°C and 70–75% relative humidity under a 16:8 light: dark regime.

#### RNA Extraction and cDNA Synthesis

RNA was extracted from the tissues (midgut, fat body, trachea, Malpighian tubules, ovary, testis, pupa, egg inner content, moth, and silk gland) and different development stages (pupa, infected larvae, moth, egg surface) of infected *A. proylei* collected from sericulture fields. The RNA was extracted using Trizol (RNAiso plus, (DSS Takara Bio India Pvt. Ltd., New Delhi, India) using a method previously described (Esvaran et al. 2019) followed by reverse transcription as per the manufacturer’s protocol (PrimeScript 1st strand cDNA synthesis kit, DSS Takara Bio India Pvt. Ltd., New Delhi, India) to generate cDNA. The cDNA was diluted 10-fold and used for normal PCR conventional analysis as well as quantitative Real time PCR (qRT-PCR) analysis.

#### Detection AnprNPV Through PCR

A PCR reaction was carried out to detect AnprNPV in viral infected tissues from different developmental stages of silkworm. The primers were designed based on the whole genome sequence information of AnprNPV (GenBank accession no. LC375539.1). In view of its consistent amplification and being indicated as a gene specific to NPV (Shantibala et al. 2018), the *Anpr*53 (*he65-like*) gene segment was selected for identification and viral quantification through PCR and qPCR. The *Anpr53 gene* product, P87 is found to be an integral part of viral capsid associated proteins. This gene family consists of several nucleopolyhedrovirus capsid protein sequences. The *Anpr*53 (*he65-like*) gene is expressed late in infection and concentrated in infected cell nuclei. BLAST analyses of *Anpr*53 indicate a 100% homology with NPVs infecting other wild silk moths such as *A. pernyi*, *Samia cynthia* (Lepidoptera: Saturniidae), and *A. yamamai* (Lepidoptera: Saturniidae).

The primers used in this study are listed in Supp Table 1 (online only). The PCR reaction mixture comprised a total volume of 10 μl composed of 1 μl of genomic DNA 0.1 μM each of forward and reverse primers, 5 μl of 2X PCR buffer, EmeraldMaster Mix (EmeraldAmp GT PCR Master Mix, DSS Takara Bio India Pvt. Ltd., New Delhi, India). The samples were subjected to the following thermal profile for amplification: 2 min at 94°C, followed by 30 cycles of [30 s at 94°C, 30 s at 55°C, and 1 min at 72°C] in a BioRad T100 Thermal cycler (Bio-Rad Laboratories India Pvt. Ltd., Gurugram, Haryana). The PCR products were resolved on a 1.2% agarose gel stained with ethidium bromide for visualization of the amplified product.

#### Real Time qPCR

To quantify the AnprNPV virus from infected *A. proylei* samples, real time PCR was carried out on extracts as described previously from the different tissues sampled. DNA amplification was carried out using the specifically designed primer (*Anpr*53) along with a non-template control. The PCR reaction mixture comprised of a total volume of 10 μl with 1 μl of the diluted cDNA, 0.1 μM each of forward and reverse primers, and 5 μl of SYBR Premix Ex Taq II (TliRnaseH Plus, DSS Takara Bio India Pvt. Ltd., New Delhi, India). The samples were subjected to the following thermal profile for amplification: 2 min at 94°C, followed by 30 cycles of (30 s at 94°C, 30 s at 57°C, and 1 min at 72°C). The PCR products were resolved on a 1.2% agarose gel and stained with ethidium bromide for visualization of the amplified product. The reactions were carried out on an Agilent Technologies Stratagene Mx3005P real time PCR machine (Agilent, Santa Clara, CA, USA) with each sample tested in triplicate, and the mean values were used for determining the viral copy number. The unknown samples were compared against a standard curve generated by serial dilutions of the *Anpr53* gene cloned into a pJET plasmid (CloneJET PCR Cloning Kit, Thermo Fisher Scientific, Waltham, MA) using the manufacturer’s instructions for the determination of copy numbers.

## Results

### Transmission Electron Microscopy (TEM)

**Fig. 1.**
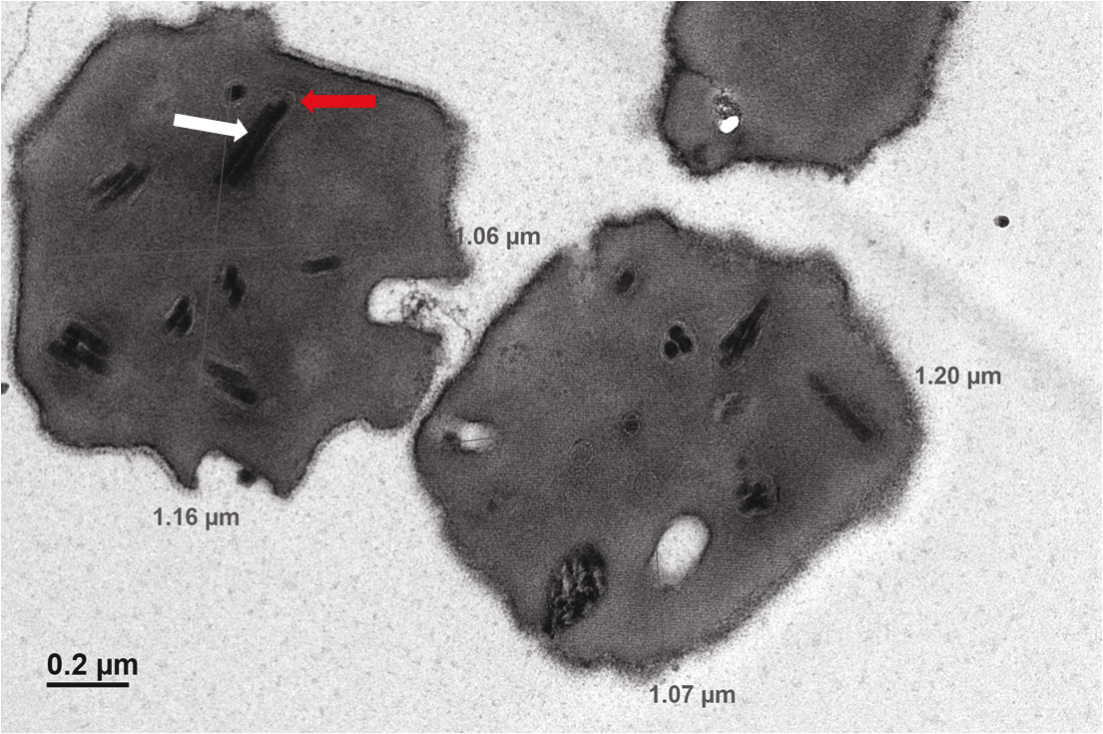
Transmission electron micrographs of AnprNPV OBs. The arrows denote the envelope surrounding the rod-shaped nucleocapsids of the virion. The nucleocapsid (white arrow) and surrounding envelope (Red arrow) of an occluded virion are indicated. The TEM image showed distinct phenotypes of virus such as single-embedded and multiple-embedded virus.

The TEM image showed the presence of occlusion derived viruses (ODVs). Further, both single and multiple rod-shaped occlusion derived virus (ODV) embedded in a single polyhedral OB was observed. This confirms that the virus purified from infected *A. proylei* silkworms are ODVs (Fig. 1). The presence of circular and rod-shaped occlusion bodies with single and multiple nucleocapsids per envelope seen in the TEM image indicates that the virus is of the multiple-enveloped phenotype, and that AnprNPV OBs spanned approximately 1.16 × 1.06 µm in size.

### Infectivity, Surface Disinfection, and Vertical Transmission of AnprNPV

The infectivity of the virus was tested by inoculating disease-free 3rd instar *A. proylei* silkworm with AnprNPV virus isolated from diseased silkworms and recording the number of days of survival and gradual progression up to death of the larvae under different dosages. As expected, larval mortality increased with an increase in the concentration of *A*nprNPV. The larval mortality after infection was observed to start from day 6, which gradually increased to 100% mortality on day 12. The LC50 value of AnprNPV in *A. proylei* was estimated to be 4.8 × 10^4^ at 95% confidence intervals (*R*^2^ > 0.95, Supp Table 2 [online only], supplementary information). The lower and upper bound LC50 values of AnprNPV in *A. proylei* were estimated to be 1.1 × 10^4^ and 2.0 × 10^5^, respectively (Supp Table 2 [online only]).

**Fig. 2.**
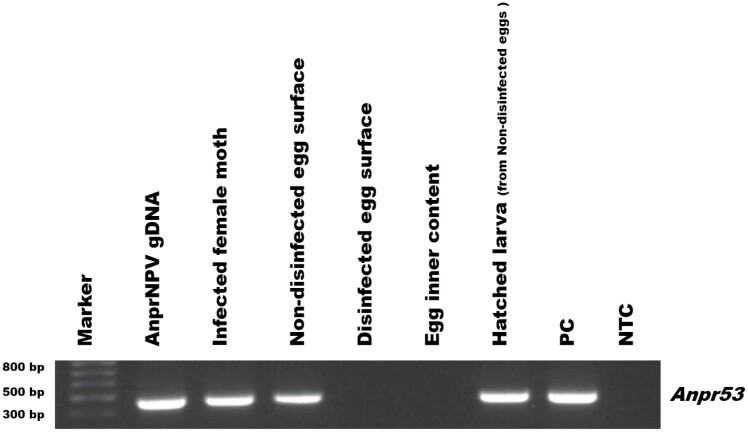
PCR test of disinfection on AnprNPV transmission in *A. proylei* females and eggs. PCR analysis of the egg surface before and after surface disinfection, surface disinfected egg inner contents, and larvae hatched from untreated eggs. The analysis was performed using primers from the *Anpr*53 gene for NPV detection, with AnprNPV DNA as positive control (PC) and a Non-Template Control (NTC).

When checked with PCR, DNA from the surface disinfected eggs did not show the presence of AnprNPV, while amplification of virus was observed in non-disinfected egg samples (Fig. 2). Further, the hatched larvae from the non-disinfected sample also displayed the presence of virus.

### Tissue Tropism

**Fig. 3.**
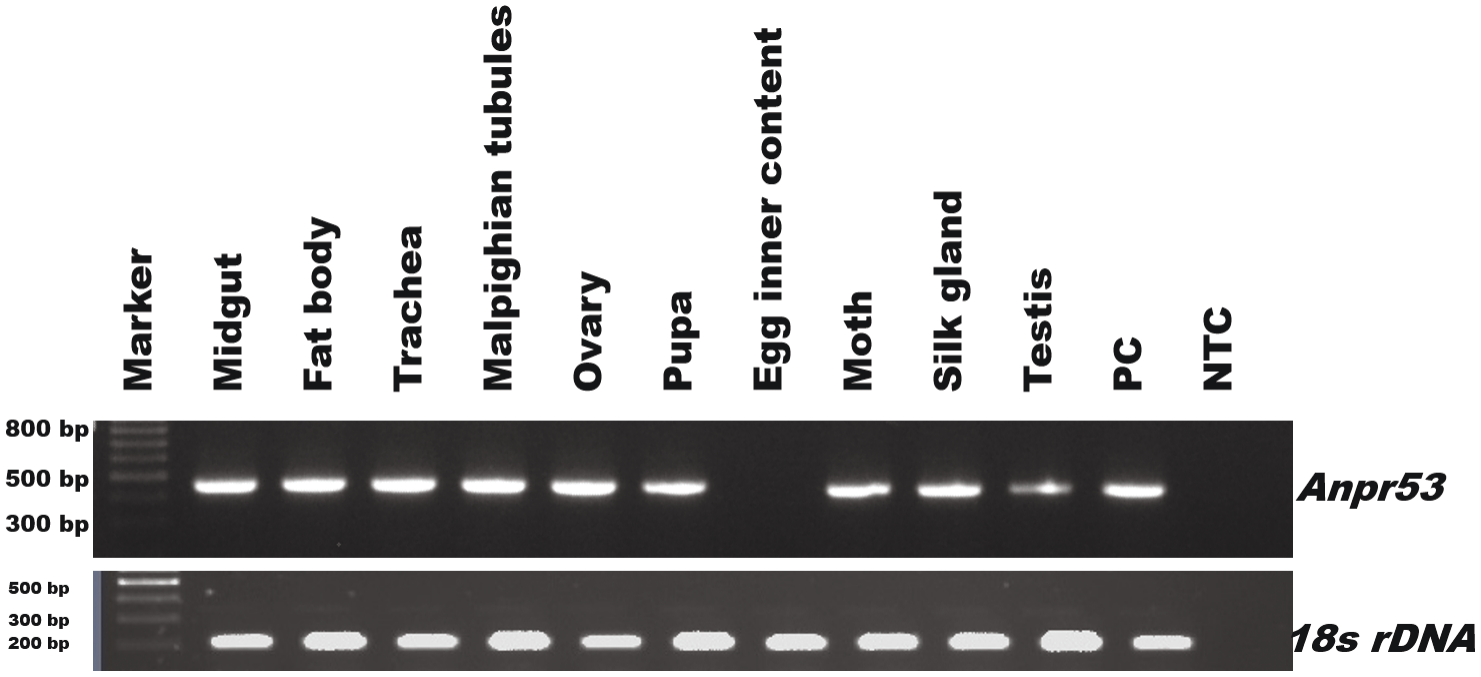
PCR analysis of AnprNPV from field collected *A. proylei* larval tissues. *Anpr*53 was used to detect the presence of NPV and 18S (a housekeeping gene) was used as an internal control along with viral DNA. PC: *A. proylei* NPV genomic DNA as positive control; NTC: a non-template control.

**Fig. 4.**
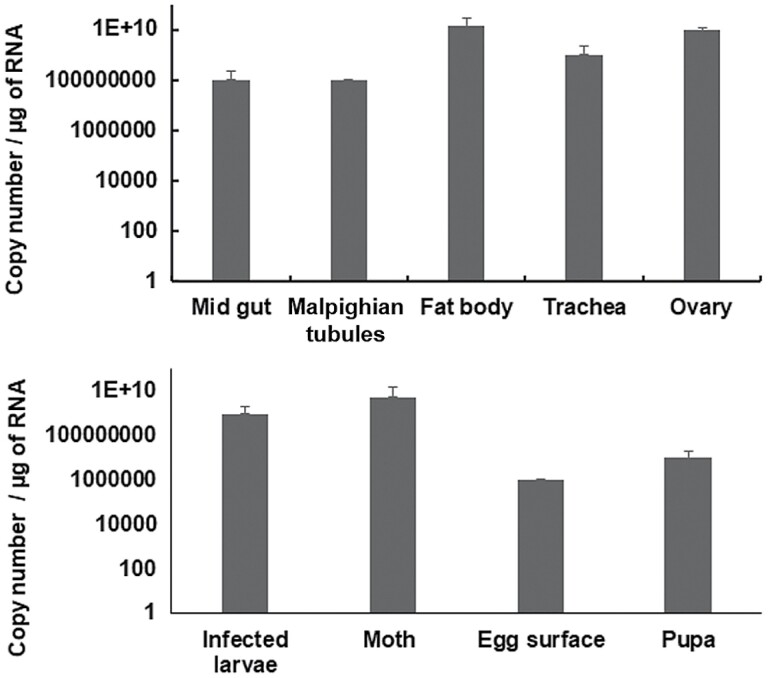
RT**-**qPCR analysis of NPV load in infected tissues at different development stages. *Anpr*53 was used to quantify the NPV and 18S (an internal reference gene) was used as an internal control. The error bars indicate the standard deviation of 3 replicates.

The presence of AnprNPV in infected *A. proylei* tissues was tested through PCR amplification. Infected tissues such as midgut, fat body, trachea, Malpighian tubules, ovary, testis, and silk gland showed positive amplification for AnprNPV (Fig. 3). The presence of virus was also noted in various development stages of infected *A. proylei* such as pupa and moth (Fig. 3). The viral copy number in each of these tissues and developmental stages was quantified using qPCR (Fig. 4). The data showed the presence of the virus and a higher copy number in the fat body (2.34 × 10^11^) and ovary (2.07 × 10^11^) in comparison with other tissues from the infected silkworm, indicating higher virus propagation in these tissues.

### Cross-Infection of AnprNPV

**Fig. 5.**
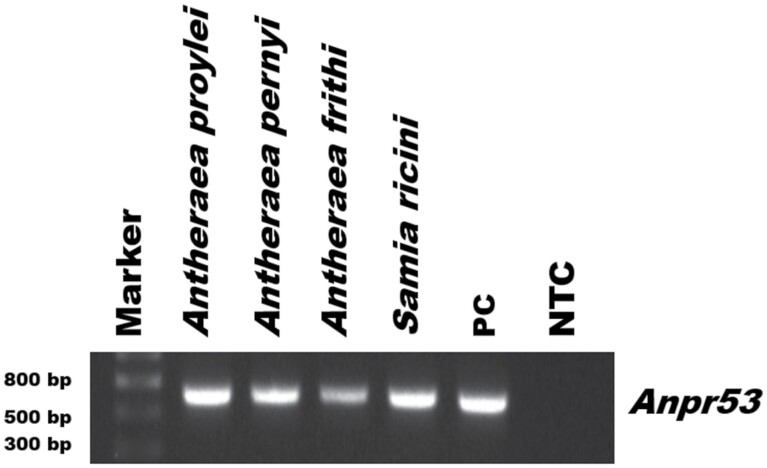
Cross-infectivity of AnprNPV to other saturniid silkworms. The purified virus particles were fed to the third instar of *A. frithi, A. pernyi* and *S. ricini* silkworms. *Anpr*53 was used to detect the presence of NPV. *A. proylei* NPV genomic DNA was used as positive control (PC); a Non-Template Control (NTC). 18S (an internal reference gene) was used as an internal control along with viral DNA. NTC without a DNA template was also used in the analysis.

Larvae of *S. ricini*, *A. pernyi*, and *A. frithi* died after exposure to the virus, indicating their susceptibility to AnprNPV infection. The mortality of *A. pernyi* and *A. frithi* was observed within 144 hr post infection (hpi), while *S. ricini* mortality was observed within120 hpi. The LC50 values of AnprNPV in *S. ricini*, *A. pernyi* and *A. frithi* were 3.1 × 10^4^, 5.4 × 10^4^, and 6.1 × 10^4^, respectively (Supp Table 2 [online only]). This corroborated the molecular detection results whereby the presence of AnprNPV was found in Saturniidae silkworms such as *S. ricini*, *A. pernyi*, and *A. frithi* (Fig. 5). Cross-infection studies confirmed that AnprNPV can infect other wild silkworms of the Saturniidae family, specifically the silkworm species used in this study.

## Discussion

A. *proylei* is one of the most prominent, viable, and commercially exploited silkworm species in India. Since it is reared outdoors, it is frequently infested with different pathogens, one of them being AnprNPV, causing tiger band disease (Shantibala et al. 2018). In the present study, TEM cross-sections revealed the OBs of AnprNPV to be approximately within the size reported in other Alphabaculovirus OBs (Harrison et al. 2018).

Unlike other *Alphabaculoviruses*, lepidopteran NPVs exhibit distinctive tissue tropism in their host insects. The majority of insect NPVs replicate only in the midgut, which is the first target tissue during oral infection. Lepidopteran NPVs, however, establish a transient infection in the midgut before affecting most of the other larval tissues (Katsuma et al. 2012). Similar kinds of observations were found with AnprNPV infection in *A. proylei*. Our RT-qPCR results showed higher replication of AnprNPV in the fat body and ovary followed by trachea (Fig. 4). Viral multiplication was also found in midgut and Malpighian tubules. These results are in support of Katsuma et al. 2012 where poor replication was reported in silk glands, midgut, and Malpighian tubule tissues of NPV infected *Bombyx mori* L. (Lepidoptera: Bombycidae) larvae. Nevertheless, the molecular mechanism involving tissue tropism of baculovirus is largely unknown and may vary between different organisms (Katsuma et al. 2012).

The results of tissue tropism indicate a systemic progression of AnprNPV infection in *A. proylei* larvae. Baculoviruses generally show a biphasic infection with two distinct phenotypes such as occlusion-derived virus (ODV) and budded virus (BV) (Jiang et al. 2021). Primary infection is caused by ODV in the midgut, while the secondary infection is caused by BV, causing systemic spread all over the host (Jiang et al. 2021). Further, AnprNPV was present in all the developmental stages of the silkworm indicating its ability to thrive and propagate throughout all these stages.

Surface disinfection is a commonly used method in the sericulture to remove surface contaminants, which ensures that newly hatched larvae are free from infection. Hence, 0.2% sodium hypochlorite (a common disinfectant) treatment was tested for eggs laid by an AnprNPV-infected mother moth. The surface disinfection with 0.2% sodium hypochlorite solution was found to be effective in removing viral particles from the surface-contaminated eggs. Further, the inner contents from the surface-treated egg samples also did not display the presence of any virus indicating the transmission route of the virus to be trans-ovum vertical transmission in nature. The hatched larvae from the non-disinfected sample also displayed the presence of virus. This might be attributed to the presence of virus on the egg surface, which may have been ingested by the larvae during eclosion.

Baculoviruses adopt a combination of horizontal and vertical transmission strategies, depending on the density of their hosts (Cory 2015, Williams et al. 2017). It is anticipated that horizontal transmission has an evolutionary advantage at high host densities, while vertical transmission is preferable in low density insect populations (Williams et al. 2017). However, it has to be noted that vertical transmission has certain limitations involving host survival and reproductive success. It has long been known that many invertebrate pathogens can be transferred directly from infected hosts to their offspring (Williams et al. 2017). In a study on spongy moth, 80% of the hatched larvae died of NPV infection due to transovum transmission of the NPV via the surface of contaminated eggs (Kukan 1999). Further, transovum and transovarial vertical transmission of BmNPV are well documented in *B. mori* silkworms (Khurad et al. 2004). The current study also indicated a vertical source of AnprNPV transmission; probably from the contaminated egg surface (transovum). This was demonstrated by the disappearance of infection after treating the egg surface with a disinfectant. However, the presence of virus was observed in the emerging offspring of untreated eggs. This phenomenon has been attributed to the feeding of newly hatched larvae on pieces of egg shell or ‘chorion’ which enables them to survive until capable of feeding on the host plants, hence ingesting any accompanying pathogen thriving on the egg surfaces. This vertical mode of transmission from infected adults to their offspring has key implications for pathogen dispersal, genetic diversity and disease persistence of AnprNPV in rearing fields. Since the virus transmits through a transovum route, we recommend precautionary measures such as decontamination of grainage houses and appliances and use of disinfectants in rearing fields to destroy the OBs of AnprNPV.

An earlier report on cross-infectivity of *A. pernyi* nucleopolyhedrovirus showed that it caused 57% mortality in larvae of *S. cynthia ricini*, whereas, *S. cynthia* nucleopolyhedrin virus did not kill the larvae of *A. pernyi* (Qian et al. 2013). To test this hypothesis, bioassays were performed with AnprNPV in Saturniidae silkworms, *A. pernyi*, *A. frithi*, and *S. ricini*. Our experimental results provided evidence for the cross infectivity of AnprNPV in all the wild saturniid silkworms tested in the study (*A. pernyi*, *A. frithi*, and *S. ricini*). This suggests that AnprNPV could be a potential pathogen to the saturniid family of silkmoth, which requires much attention to develop early diagnostic and prophylactic measures to control tiger band disease in the *A. proylei* rearing fields. The PCR and qRT-PCR techniques used in this study can be utilized to screen field samples for early identification of the pathogen. Further research with respect to baculovirus infection of silkworms can help understand more fully which host pathogen interactions are critical, and the immune response of the host to infection.

This is a first report where the AnprNPV disease occurrence, its etiology along with transmission has been described. Based on the study data, an egg surface decontamination technique was devised which could remove the virus from the surface of the eggs. Currently, the method has been widely popularized in the field to reduce the incidence of diseases and improve the remuneration of farmers (unpublished).

## Supplementary Material

ieac057_suppl_Supplementary_Files
