# Supplementary material for: Investigation on Pathological Aspects, Mode of Transmission, and Tissue Tropism of Antheraea proylei Nucleopolyhedrovirus Infecting Oak Tasar Silkworm
Source: J Insect Sci. 2022 Oct 7;22(5):9. doi: 10.1093/jisesa/ieac057 (PMC9543400; doi:10.1093/jisesa/ieac057)
Supplement: ieac057_suppl_Supplementary_Files [file ieac057_suppl_supplementary_files.docx]

**Supplementary files**

Fig. S1: Healthy (A), tiger band diseased (B) and highly infected dead cadaver (C) of oak tasar silkworm, *Antheraea proylei* Jolly (Lepidoptera: Saturniidae)*.* The tiger disease is characterized by appearance of black bands/strips across the larval body (B & C).


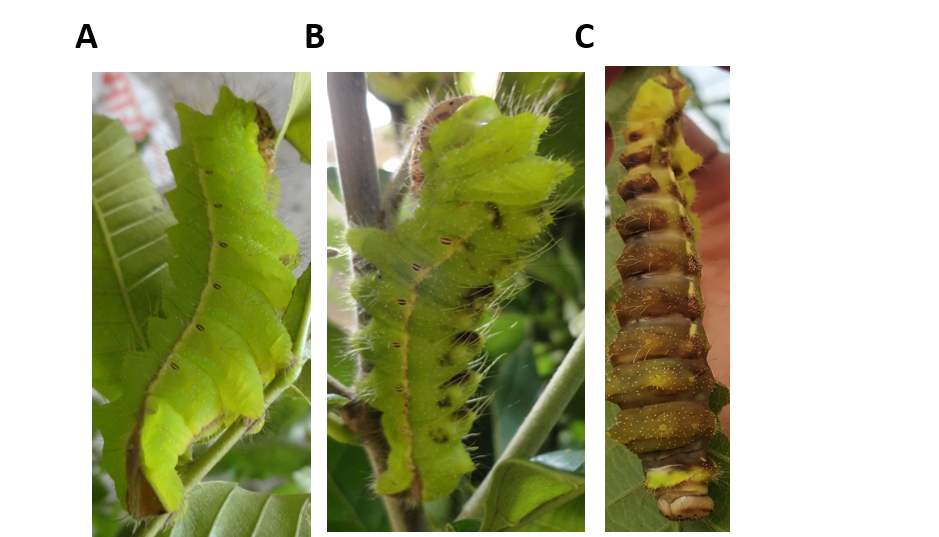


**Table S1: Primers used in this study**

| **Sl No.** | **Primer name** | **Sequence (5’>3’)** | **Amplicon Size** | **T_m_ (ᵒC)** | **GenBank accession No.** |
| --- | --- | --- | --- | --- | --- |
| 1. | *Anpr*53 qPCR F | GCAGCAATTTCCTTCCAAAG | 193 | 55.3 | LC375539.1 |
|  | *Anpr*53 qPCR R | TCTGTTGACGCTCTTGTTGG | 193 | 57.3 |  |
| 2. | *Anpr*53 F | TCTGTTGACGCTCTTGTTGG | 454 | 57.3 | LC375539.1 |
|  | *Anpr*53 R | TGTGGTACGTTGCCGAGATA | 454 | 57.3 |  |
| 3. | 18S rDNA F | ATGACGGAAGGGCACCACCAG | 131 | 61.9 | LR594608.1 |
|  | 18S rDNAR | GCACCACCACCCACGGAATCG | 131 | 66.67 |  |

**Table S2: LC-50 values of AnprNPV in *Antheraea proylei, Samia ricini, Antheraea pernyi*, and *A. frithi.* The LC-50 values were calculated by Probit analysis with Finney’s Probit analysis spreadsheet calculator (**[**https://probitanalysis.wordpress.com/**](https://probitanalysis.wordpress.com/)**).**

| **Organism** | **95% Confidence Limits for LC-50 (OB/ml)** | | | **Slope** | **Intercept** | **R^2^** | **Chi-test (χ2) Sig** |
| --- | --- | --- | --- | --- | --- | --- | --- |
|  | LC-50 | Lower bound | Upper bound |  |  |  |  |
| *Antheraea proylei* | 4.8 x 10^4^ | 1.1 x 10^4^ | 2.0 x 10^5^ | 0.56 | 2.36 | >0.95 | >0.95 |
| *Samia ricini* | 3.1 x 10^4^ | 7.08 x 10^3^ | 1.4x 10^5^ | 0.54 | 2.56 |  |  |
| *Antheraea pernyi* | 5.4 x 10^4^ | 1.2 x 10^4^ | 2.3 x 10^5^ | 0.56 | 2.32 |  |  |
| *Antheraea frithi* | 6.1 x 10^4^ | 1.4 x10^4^ | 2.5 x 10^5^ | 0.56 | 2.28 |  |  |
